# Trichloroacetic Acid Ingestion: Self-Harm Attempt

**DOI:** 10.1155/2017/3701012

**Published:** 2017-09-10

**Authors:** E. R. Black

**Affiliations:** Department of Psychiatry, Southern Illinois University, Springfield, IL 62702, USA

## Abstract

**Objective:**

Trichloroacetic acid (TCAA), or trichloroethanoic acid, is a chemical analogue of acetic acid where three methyl group hydrogen atoms are replaced by chlorine. TCAAs are also abbreviated and referred to as TCAs, causing confusion with the psychiatric antidepressant drug class, especially among patients. TCAAs exist in dermatological treatments such as chemical peels or wart chemoablation medication. TCAA ingestion or overdose can cause gastric irritation symptoms including vomiting, diarrhea, or lassitude. This symptomatology is less severe than TCA overdose, where symptoms may include elevated body temperature, blurred vision, dilated pupils, sleepiness, confusion, seizures, rapid heart rate, and cardiac arrest. Owing to the vast difference in symptoms, the need for clinical intervention differs greatly. While overdose of either in a self-harm attempt can warrant psychiatric hospital admission, the risk of death in TCAA ingestion is far less.

**Case Report:**

A patient ingested TCAA in the form of a commercially available dermatological chemical peel as a self-harm attempt, thinking that it was a more injurious TCA.

**Conclusion:**

Awareness among physicians, particularly psychiatrists, regarding this relatively obscure chemical compound (TCAA) and its use by suicidal patients mistakenly believing it to be a substance that can be significantly more lethal (TCA), is imperative.

## 1. Introduction

Chemexfoliation (chemical peeling) is used to obtain both therapeutic and cosmetic benefits [1]. TCAA, formally used as an herbicide, can be used for such dermatologic purposes and is widely available without prescription, including via Internet retail sources. While exceedingly rare in overdose, TCAA's similarity in name with TCAs can attract psychiatric patients who desire self-harm, mistakenly believing TCAAs have the same lethality in overdose. It is also true that TCAAs can even be abbreviated as TCAs, causing further misperception [2]. Fortunately, TCAAs are not significantly absorbed into the bloodstream and therefore do not produce significant systemic complications other than vomiting and diarrhea even in higher concentrations of herbicide TCAAs [3]. However, a form of acute poisoning via ingestion can manifest. Symptoms of burning pain in mouth, pharynx, and abdomen followed by vomiting and diarrhea with dark blood are typical of overdose. Asphyxia can even occur from edema of the glottis. Dizziness and weakness may occur as well, with reports of a 6–8-hour latent period followed by pulmonary edema. In acute poisoning, hemoconcentration may be indicated by rise in red blood cell count and hematocrit as with other acid and acid-like corrosives [4].

This report describes a patient who previously developed mild symptoms related to a TCAA overdose in a self-harm attempt while believing it to contain a TCA. The objective is to draw attention that this type of overdose which, while rare, may warrant awareness of the commercial availability of TCAAs for patients and the possibility of their use in self-harm behaviors.

## 2. Case Report

A 19-year-old female was evaluated at the Department of Adult Psychiatry Outpatient Clinic with complaints of lack of enjoyment of pleasurable activities, sleep disturbances, suicidal thoughts, and self-induced injury at times of high stress via superficial cutting behaviors on her forearms. These symptoms were in association with ongoing family conflict with her mother and her sister with whom she lived.

At the psychiatric evaluation, she expressed that her self-esteem was strongly affected by her turbulent family relationships, and she seemed extremely unhappy. She reported superficial cutting behaviors at least 2 times a week, usually in an impulsive fashion. Laboratory evaluations including hemogram, liver function tests, total protein, vitamin B12, folic acid, T3, T4, and TSH were within normal limits. Baseline psychiatric evaluation with the Beck Depression Inventory (BDI-II) revealed scores of 21 (moderate depression) [5]. According to clinical evaluation as well as DSM-V criteria, the patient was diagnosed with major depressive disorder and borderline personality disorder, and she was started on citalopram 20 mg by mouth per day [6]. Cognitive behavioral therapy focusing on negative cognitions was also initiated. Partial response to treatment was observed at the 12th week with reduction of BDI-II score to 12 (mild mood disturbance).

While she was under follow-up at the Department of Adult Psychiatry Outpatient Clinic, she admitted to a prior ingestion of a portion of a small bottle (less than 30 milliliters per her description of the product packaging) of a commercially available chemical skin exfoliator containing TCAA. This consumption was impulsive per her description and occurred after an argument with her mother. While such products available over the counter can typically range from 8% to 30% TCAA ingredient which determines strength, she did not recall what percentage of TCAA was in the product [7]. She had purchased the TCAA peel to brighten and even out her skin tone by reducing the appearance of hyperpigmentation (acne marks and freckles). She believed the product to also contain antidepressant TCA, since the product was marketed per her report as a “TCA peel,” which is often done in the industry. She knew that TCA antidepressants can be extremely damaging or even fatal in overdose which was her intent. TCA effects in overdose are well documented in the literature [8]. Her symptoms after ingestion were burning pain on her lips and in her mouth, which frightened her somewhat, preventing her from consuming more of the product. She also complained at the time of mild abdominal discomfort followed by an episode of vomiting. Fortunately no other symptoms were reported and all of the symptoms resolved within a few hours. She was asymptomatic at the time of reporting this event to the outpatient clinic.

## 3. Discussion

In clinical psychiatric practice, treating a patient with impulsivity related to poor coping skills and self-harm desire related to borderline personality disorder can be challenging. Less common self-harm attempts via ingestion of a wide array of chemical substances and even objects can occur with these patients. Foreign body ingestion cases have been well documented in the literature [9, 10]. Self-harm attempts, including such consumption of chemical substances, can be fueled by patient misperceptions and misunderstandings regarding the content of the substances and the desired effects. Some patients will believe that a relatively benign substance may do extreme or lethal harm to themselves, as in this case.

The differentiation between various rare substances, including herbal, homeopathic, and cosmetic products available on the market, can also be extremely difficult for physicians. The resulting consequences from misuse of such substances can pose further problems in the diagnosis and management of patients who ingest these products in self-harm or suicide attempts.

In the present case, although response was achieved through psychological and pharmacological treatment with regard to the patient's mental health diagnoses, the report of a prior impulsive self-harm ingestion of a relatively rarely encountered substance was cause for alarm. Exploration as to the patient's motives for this ingestion was warranted and thus uncovered that she misunderstood the nature of the product. She confused a TCAA skin peel with a traditional antidepressant TCA. This discussion led to further clinical characterization of the patient's perceived lethality of the substance. Her previous desire for self-harm was evident regardless of the substance's actual ability to harm. In conclusion, physicians and, especially, psychiatrists should remain mindful of the relatively obscure chemical compounds that may be used in overdose, especially by suicidal patients who believe the substance to be of increased lethality.
